# Delivery of human natural killer cell-derived exosomes for liver cancer therapy: an in vivo study in subcutaneous and orthotopic animal models

**DOI:** 10.1080/10717544.2022.2118898

**Published:** 2022-09-06

**Authors:** Ho Yong Kim, Hyun-Ki Min, Hyeong-Woo Song, Ami Yoo, Seonmin Lee, Kyu-Pyo Kim, Jong-Oh Park, You Hee Choi, Eunpyo Choi

**Affiliations:** a Korea Institute of Medical Microrobotics, Buk-gu, Gwangju, Republic of Korea; b Department of Oncology, Asan Medical Center, University of Ulsan College of Medicine, Songpa-Gu, Seoul, Republic of Korea; c School of Mechanical Engineering, Chonnam National University, Buk, Gwangju, Republic of Korea

**Keywords:** Natural killer cells, exosomes, hepatocellular carcinoma, liver cancer therapy

## Abstract

Exosomes are nanosized extracellular vesicles secreted by various cell types, including those of the immune system, such as natural killer (NK) cells. They play a role in intercellular communication by transporting signal molecules between the cells. Recent studies have reported that NK cell-derived exosomes (NK-exo) contain cytotoxic proteins-induced cell death. However, the characteristics and potential functions of NK-exo, especially for the liver cancer are poorly understood. In this study, we investigated the anti-tumor effects of NK-exo in the primary liver cancer, hepatocellular carcinoma (HCC), using the orthotopic and subcutaneous tumor model. We found that NK-exo expressed both typical exosomal markers (e.g. CD63, CD81, and Alix) and cytotoxic proteins (e.g. perforin, granzyme B, FasL, and TRAIL). NK-exo were selectively taken up by HCC cells (e.g. Hep3B, HepG2, and Huh 7). Interestingly, Hep3B cells induced the highest cytotoxicity compared with HepG2 and Huh7 cells, and substantially enhanced the apoptosis by NK-exo. Furthermore, we demonstrated that NK-exo inhibited the phosphorylation of serine/threonine protein kinases (e.g. AKT and ERK1/2), and enhanced the activation of specific apoptosis markers (e.g. caspase-3, -7, -8, -9, and PARP) in Hep3B cells. NK-exo also exhibit the active targeting ability and potent therapeutic effects in both orthotopic and subcutaneous HCC mouse models. Overall, these results suggest that NK-exo indicate strong anti-tumor effects in HCC, which are mediated by novel regulatory mechanisms involved in serine/threonine kinase pathway-associated cell proliferation and caspase activation pathway-associated apoptosis.

## 1. Introduction

Hepatocellular carcinoma (HCC) is the most common form of primary liver cancer and the third most common cause of cancer-related deaths worldwide (Balogh et al., 2016; Craig et al., 2020). HCCs tend to be inoperable owing to the co-existence with other liver diseases and delay in diagnosis resulting from the lack of pain. However, the precise underlying mechanisms and overall prognosis of HCC are not fully understood. For several decades, immunotherapeutic approaches have been unsuccessful in HCC treatment. Immune-checkpoint inhibitors (ICIs) such as programmed cell death (PD-1) and its ligand (PD-L1), which act by reversing immunosuppression have recently been shown to be effective in the treatment of HCC (Inarrairaegui et al., 2018). Tyrosine-protein kinase inhibitors (e.g. sorafenib, regorafenib, lenvatinib, and nivolumab)-based liver cancer therapies are considered the most efficient targeted drugs for advanced HCC (Cheng et al., 2009; Yau et al., 2009; El-Khoueiry et al., 2017). Nevertheless, the response rates to PD-1/PD-L1 inhibitors, in combination with both nivolumab and sorafenib for the treatment of HCC are still less than 20%. Hence, there is an urgent need for the development of new therapeutic strategies based on anti-tumor immune responses for the treatment of HCCs.

Natural killer (NK) cells are innate immune lymphoid cells that play a central role as effectors and regulators of the immune system. NK cells act via one of two major cytotoxic pathways (Shimasaki et al., 2016). The first is an intrinsic apoptosis pathway; this involves small granules contained membrane disrupting protein, such as perforin, which cooperate with serine proteases, including granzyme B, to trigger sequential molecular events resulting in the release of cytochrome c. Perforin secreted from NK cells, forms pores in the target cell membrane, allowing granzyme B to enter the target cell and induce cell death (Voskoboinik et al., 2006). The second is an extrinsic apoptosis pathway; this involves cell death ligands, including the Fas ligand (FasL) and tumor necrosis factor (TNF)-related apoptosis-inducing ligand (TRAIL), acting via their cognate receptors (Fas and TRAIL-R1/TRAIL-R2), respectively. Death ligands (e.g. FasL and TRAIL) on NK cell surface, upon binding to the death receptors (e.g. Fas and TRAIL-R1/TRAIL-R2) on target cells, activate NK cell cytotoxicity, thereby inducing caspase-dependent apoptotic cell death (Warren & Smyth, 1999; Wang et al., 2012; Feng et al., 2019). The action of death ligands, FasL and TRAIL, requires transient, multiple NK cell-target conjugations to activate caspase signaling effectors (e.g. caspase-3, -7, -8, and -9) and poly(adenosine diphosphate [ADP]-ribose polymerase) (PARP). The mitogen-activated protein kinase (MAPK) pathway, involving extracellular signal-regulated kinase 1/2 (ERK 1/2), and the phosphatidylinositol-3-kinase (PI3K) pathway, involving protein kinase B (AKT), play a critical role in the effects of NK cells on cancer cell survival and proliferation (Jiang et al., 2000). The MAPK-ERK pathway promotes cell proliferation, whereas the PI3K-AKT pathway is associated with cell survival. To control tumor survival and proliferation, NK cells regulate the cytotoxic activities of various tumor growth factors via the AKT and ERK signaling pathways (Yang et al., 2017; Jin et al., 2019).

Exosomes are extracellular vesicles of 30–150 nm that are secreted and taken up by all cell types (Thery et al., 2009). Exosomes were initially thought to be ‘garbage bags’ used to remove unwanted cellular constituents. However, exosomes play a critical role in metastasis (Zhao et al., 2018). They are involved in intracellular communication, required for the mediation of cellular functions, including survival, proliferation, and apoptosis (Greening et al., 2015). Exosomes are being explored as sensitive biomarkers for the diagnosis of cancers. Exosomes contain characteristic compositions of genetic molecules (e.g. DNA, RNA, proteins, and lipids) and include membrane transport proteins (Annexins and Flotillin), plasma membrane proteins (tetraspanins: CD9, CD63, CD81, and CD82), proteins associated with multivesicular bodies [Alix and tumor susceptibility gene 101 (TSG101)] (Thery et al., 2006; Thery, 2011; Raposo & Stoorvogel, 2013; Tkach & Thery, 2016). Specifically enriched proteins (e.g. CD9, CD63, CD81, and CD82) are widely used as exosome markers. Exosomes derived from each cell type may exhibit a unique protein characterization and regulate functions depending on the origin of cells (Kim et al., 2017).

Many studies have focused on the potential role of exosomes in cancer. An understanding of the characteristics and potential functions of exosomes is essential for elucidating their biological roles and for investing in their potential clinical use (Chaput et al., 2005; Kalluri, 2016; Kalluri & LeBleu, 2020). Dendritic cell-derived exosomes (DC-exo) have been shown to cause immune activation and have been used in the treatment of tumors (Pitt et al., 2016). Mesenchymal stem cell-derived exosomes (MSC-exo) can act as potent therapeutic vehicles, mediating cartilage repair by enhancing proliferation, attenuating apoptosis, and increasing immune reactivity (Zhang et al., 2018; Xunian & Kalluri, 2020). Notably, NK cell-derived extracellular vesicles (NK-EVs) including exosomes typically contain cytotoxic proteins (e.g. perforin, granzyme B, FasL, and TRAIL), originating from NK cells and induce anti-tumor activity against various cancers through the activation of caspase-dependent pathways (Jong et al., 2017; Neviani et al., 2019; Sun et al., 2019; Wu et al., 2019; Zhu et al., 2019). NK cell-derived exosomes (NK-exo), in particular, exhibit high tumor cell-killing potential through the regulation of cytotoxic activity against several cancer types (e.g. neuroblastoma, melanoma, and breast carcinoma) (Lugini et al., 2012; Jong et al., 2017; Shoae-Hassani et al., 2017; Zhu et al., 2017; Wang et al., 2019; Di Pace et al., 2020). However, the characteristics and potential functions of NK-exo as anti-tumor effectors for the liver cancer, especially HCC are remain unclear.

In the present study, we aim to investigate whether NK-exo exhibit the biological roles, such as tumor targeting ability and anti-tumor effects against HCC, through regulation of cell survival, proliferation, and apoptosis *in vitro* and *in vivo*. Here, we were the first to report that NK-exo enhanced the cytotoxicity and apoptosis in HCC cells. The novel signaling mechanisms involved in specific serine/threonine kinase pathway (including AKT and ERK1/2) were found to regulate cell survival and proliferation, whereas the apoptosis pathways (including perforin, granzyme B, FasL, and TRAIL) were found to mediate cell death *in vitro* and *in vivo*. Furthermore, NK-exo also induced significant therapeutic effects via strong tumor targeting and anti-tumor effects in an orthotopic and subcutaneous HCC mouse models. NK-exo regulated the active targeting and cell death of HCC by two distinct mechanisms, including membrane fusion (perforin and granzyme B) and ligand–receptor interaction (FasL and TRAIL). First, perforin secreted from NK-exo formed pores on the HCC cell membranes and allowed granzyme B to enter the HCC cells. Second, the cell death ligands (FasL and TRAIL) on NK-exo surface bound to the death receptors on HCC cells and activated NK-exo cytotoxicity, thereby to induce caspases-dependent apoptosis. Through the aforementioned mechanisms, NK-exo can have a targeting ability in solid tumors such as HCC. Our findings provide evidence for the anti-tumor activity of NK-exo and reflect their potential as an immunotherapeutic strategy for the treatment of solid tumors, including HCC.

## 2. Materials and methods

### 2.1. Cell culture

Hep3B, HepG2, and Huh7 cells were purchased from the Korean Cell Line Bank (Seoul, Korea) and cultured in Dulbecco’s minimum essential medium (Corning, NY, USA), supplemented with 10% fetal bovine serum (FBS; Corning) and 1% penicillin–streptomycin (P/S; Corning). Human NK-92 cells were purchased from the American Type Culture Collection (Manassas, VA, USA) and cultured in α-minimum essential medium (Corning), which was composed of 12.5% FBS (Corning), 12.5% horse serum (HS; Sigma-Aldrich, St. Louis, MO, USA), 1% P/S (Corning), 0.2 mM Myo-inositol (Sigma-Aldrich), 0.1 mM 2-mercaptoethanol (Sigma-Aldrich), 0.02 mM folic acid (Sigma-Aldrich), 10 ng/mL Interleukin (IL)-2 (Miltenyi Biotec, Bergisch Gladbach, Germany), and 20 ng/mL IL-15 (Miltenyi Biotec) activating cytokines. Normal human liver THLE-2 cells were obtained from Dr. KP Kim (Asan Medical Center, Seoul, Korea) and were cultured in bronchial epithelial cell growth medium (BEGM Bullet Kit; Lonza, Basel, Switzerland) as per the manufacturer’s protocol. All cells were cultured at 37 °C with 5% CO_2_.

### 2.2. Exosome isolation

NK-92 cells (1 × 10^7^) were cultured in conditional medium, supplemented with exosome-depleted FBS or HS, prepared by ultracentrifugation (Himac CP100NX; HITACHI, Tokyo, Japan) at 120,000*g* for 18 h at 4 °C (Shelke et al., 2014). After 72 h, the cultured media were sequentially centrifuged at 300*g* for 5 min, 2,000*g* for 15 min, and 10,000*g* for 30 min to remove residual cells, dead cells, and cell debris. The supernatant was concentrated on a tangential flow filter system (TFF-Easy; HansaBioMed Life Sciences, Tallinn, Estonia) and was again subjected to ultracentrifugation at 100,000*g* for 90 min to pellet exosomes (Lobb et al., 2015). The isolated exosomes were resuspended in 1 mL phosphate-buffered saline (PBS; Corning). Size and density were measured by dynamic light scattering (DLS) and nanoparticle tracking analysis (NTA).

### 2.3. Transmission electron microscopy

Transmission electron microscopy (TEM) was used to evaluate the morphology of the isolated NK-exo. The NK-exo were fixed using a 2% paraformaldehyde solution and dropped onto a Formvar/carbon-coated TEM grid (Agar Scientific, Stansted Mountfitchet, UK). After 20 min, the fixed grid was rinsed with a small drop of PBS for 10 s. The grids were then dried for 10 min and observed using a transmission electron microscope (JEM-1400; JEOL, Tokyo, Japan) at an acceleration voltage of 80 kV. Digital images were captured using a Mega View Camera (EMSIS, Germany).

### 2.4. Dynamic light scattering

Zetasizer Nano ZS (Malvern Ins., Malvern, UK) equipped with a 633 nm laser was used for exosome particle size analysis by DLS. Samples were contained in a square cuvette prior to analysis (1 mL, 1:100 dilution with PBS).

### 2.5. Nanoparticle tracking analysis

The size and number of the isolated NK-exo (10 μL, 1:100 dilution with PBS) were detected by NTA using the Nanosight LM10 system (Malvern Ins., Worcestershire., UK) equipped with a 405 nm laser. The data were collected and analyzed using an NTA software (Nanosight version 3.1; Malvern).

### 2.6. Western blot analysis

Cell lysates and isolated exosomes were lysed with radioimmunoprecipitation assay (RIPA) buffer (GenDEPOT, Barker, TX, USA) containing 1% protease inhibitor (GenDEPOT) for 30 min on ice. The protein lysates were separated by sodium dodecyl sulfate–polyacrylamide gel electrophoresis (SDS-PAGE) using 10%–12% gels, transferred onto polyvinylidene difluoride membranes (PVDF; Millipore, Billerica, MA, USA), and blocked with 5% skim milk (Sigma-Aldrich) in 1× Tris-buffered saline with 0.1% Tween-20 (TBST; pH 7.4, GenDEPOT) at 25 °C for 1 h. The membranes were then subsequently incubated with specific primary antibodies and horseradish peroxidase (HRP)-conjugated secondary antibodies (Santa Cruz Biotechnology, Dallas, TX, USA). The blots were analyzed using the Amersham^TM^ Imager 6000 imaging system (GE Healthcare, Chicago, IL, USA). The ImageJ software (Ver.1.53e, NIH, Bethesda, MD, USA) was used for the quantitative analysis of the western blots. The following antibodies and reagents were used: CD63 (ab134045), CD81 (ab59477), p-AKT (ab81283), and AKT (ab179463) (1:1,000 dilution) were purchased from Abcam (Cambridge, MA, USA); perforin (sc-373943), granzyme B (sc-8022), TRAIL (sc-8440), FasL (sc-33716), Alix (sc-53540), and GAPDH (sc-97724) (1:1,000 dilution) were obtained from Santa Cruz Biotechnology (Dallas, TX, USA). p-ERK1/2 (no. 9101), ERK1/2 (no. 4695), cleaved-caspase 3 (no. 9664), cleaved-caspase 7 (no. 8438), cleaved-caspase 8 (no. 8592), cleaved-caspase 9 (no. 9505), cleaved-PARP (no. 5625), caspase 3 (no. 9662), caspase 7 (no. 12827), caspase 8 (no. 4790), caspase 9 (no. 9508), PARP (no. 9532), cytochrome c (no. 4280), and β-actin (no. 4970) (1:1,000 dilution) were purchased from Cell Signaling Technology (Danvers, MA, USA); AKT inhibitor (no. 124005) and ERK1/2 inhibitor (U0126, no. 662005) were obtained from Calbiochem (San Diego, CA, USA).

### 2.7. Cellular uptake assay

To analyze the cellular uptake of NK-exo into Hep3B, HepG2, Huh7, and THLE-2 cells, resuspended NK-exo were labeled with the green fluorescent dye PKH67 (Green Fluorescent Cell Linker Mini Kits; Sigma-Aldrich), as per the manufacturer’s instructions. Hep3B, HepG2, Huh7, and THLE-2 cells were seeded on a coverslip (1 × 10^4^ cells) and co-incubated with PKH67-labeled NK-exo (50 μg) at 37 °C for 10 h. The incubated cells were washed thrice with PBS and stained with Hoechst 33258 (Sigma-Aldrich) to visualize the nuclei. After mounting with a coverslip using Aqua-Poly/Mount (Polysciences, Inc., Warrington, PA, USA), the cellular uptake of NK-exo was observed using a confocal laser scanning microscope (LSM 880; Carl Zeiss, Oberkochen, Germany).

### 2.8. Cytotoxicity analysis

To determine the cytotoxic activity of NK-exo against Hep3B, HepG2, and Huh7 cells, we performed a lactate dehydrogenase (LDH) assay using a CyQUANT LDH cytotoxicity assay kit (Thermo Fisher Scientific), as per the manufacturer’s protocol. The release of LDH into the cell culture medium is an indicator of cytotoxicity and cytolysis. In brief, Hep3B, HepG2, and Huh7 cells were seeded in 96-well plates (1 × 10^4^ cells/well) and co-cultured with NK-exo in a dose- and time-dependent manner. At the desired time points, cultured Hep3B, HepG2, and Huh7 cells were centrifuged at 600*g* for 5 min to settle the cellular debris in the media. After centrifugation, 50 μL of the collected media was transferred to a new plate and added to 50 μL of the LDH reaction mixture. After incubating at room temperature for 30 min in the dark, the reaction was stopped by adding 50 µL of the LDH stop solution. LDH activity was determined at 490 nm and 680 nm using a microplate reader (Varioskan Flash; Thermo Fisher Scientific).

### 2.9. Cell viability analysis

To evaluate the cell viability of Hep3B, HepG2, Huh7, and THLE-2 cells in the presence of NK-exo, we performed a cell counting kit 8 (CCK-8) assay (Dojindo Molecular Technologies, Tokyo, Japan) according to the manufacturer’s instructions. Briefly, cells were seeded in 96-well plates (4 × 10^4^ cells/well) and co-cultured with NK-exo to evaluate in a time- and dose-dependent manner. Then, 10 μL of the CCK-8 solution was added to each well and the plates were incubated at 37 °C for 2 h. After incubation, the absorbance was measured at 450 nm using a microplate reader (Thermo Fisher Scientific). The viability rate of the co-cultured cells was calculated relative to that of control cells.

### 2.10. Apoptosis analysis

The effects of NK-exo on cell apoptosis were determined using fluorescein isothiocyanate (FITC)-conjugated Annexin V (Annexin V-FITC) and propidium iodide (PI) in an apoptosis detection kit (Sigma Aldrich), according to the manufacturer’s instructions. Briefly, Hep3B and THLE-2 cells (1 × 10^6^ cells/well) were seeded and co-cultured with different doses of NK-exo for different durations. Cells were collected and suspended in 500 μL of Annexin V-FITC-binding buffer. Then, the resuspended cells were stained with 5 μL of Annexin V-FITC, followed by 10 μL of PI staining solution at room temperature for 15 min in the dark. Within 2 h, the stained cells were analyzed using a magnetic-activated cell sorting flow cytometer (MACSQuant VYB; Miltenyi Biotec). Independent experiments were performed three times.

### 2.11. In vivo tumor xenograft mouse models

The protocols for all animal experiments were approved by the Institutional Animal Care and Use Committee (IACUC) of Chonnam National University (CNU IACUC-YB-2020-73 for orthotopic xenograft model and CNU IACUC-YB-2021-31 for subcutaneous xenograft model). BALB/c nude, 6-week-old, male mice were purchased from Orient Bio Inc. (Seongnam-si, Korea). The animals were housed in an environment-controlled cage system (Mouse Individually Ventilated Cage Rack System; LAB&Bio, Changwon-si, Korea). HCC tumor models were established via two different techniques. The subcutaneous xenograft model was established by subcutaneously injecting Hep3B cells (1 × 10^7^ cells/100 μL) into the right back of BALB/c nude mice. The orthotopic xenograft model was generated by injecting Hep3B cells (2 × 10^6^ cells/50 μL) directly into the liver of BALB/c nude mice. HCC was detected approximately 2 weeks later.

### 2.12. In vivo targeting analysis

To evaluate the targeting effects of NK-exo in HCC-bearing mice, we subcutaneously inoculated 100 μL of Hep3B cells (1 × 10^7^ cells) into the right back of the mice. NK-exo were labeled with DiD (red fluorescent dye, V22887; Thermo Fisher Scientific) as per the manufacturer’s instructions. After 2 weeks, the HCC-bearing mice were intravenously administered DiD-labeled NK-exo (500 μg) and were sacrificed after 24 h. The target tumor and main organs (the heart, liver, spleen, kidney, and lung) were collected and analyzed using an *in vivo* imaging system (IVIS; NightOWL II LB 983, Berthold Technologies, Wellesley, MA, USA). The fluorescence intensity of NK-exo in each tissue was also measured *ex vivo*.

### 2.13. In vivo anti-tumor activity

To investigate the anti-tumor activity of NK-exo in HCC tumor growth, the mice were inoculated with 50 µL of Hep3B cells (2 × 10^6^ cells) directly into the liver. After 2 weeks, HCC tumors were detected and the mice were divided into five groups (five mice per group). HCC-bearing mice were administered PBS (control) and different doses of NK-exo via intravenous injection, once every 2 days for 12 days. To evaluate the therapy effects of NK-exo to suppress tumors, the HCC-bearing mice were monitored until 12 days and then sacrificed. The livers were resected. The body weights were calculated from 0 day to 12 day. Tumor weights were determined at the end of experiment and calculated by the isolated HCC tumor weight from the total liver. The tumor inhibition rate was calculated according to: [1 – (weight of tumor in NK-exo treated group/weight of tumor in PBS control group)] × 100 (%).

Next, to observe the specific antigen expression of NK-exo in HCC tumor tissues, liver tumors were isolated and fixed in 10% formalin for histological analysis. The formalin-fixed liver tumors were embedded in paraffin and sectioned into 8 μm slices using a microtome (RM2255; Leica Biosystems, Wetzlar, Germany). After deparaffinization and rehydration, some tissue slices were stained with hematoxylin–eosin (H&E) and the others were used for immunohistochemical (IHC) analysis. To unmask the crosslinked antigens, the tissue slides were retrieved by heating in citrate buffer (pH 6.0) using the microwave for 20 min. Then, tissue sections were blocked by exposing them to 5% bovine serum albumin for 1 h. After blocking, the tissue slices were incubated with anti-cleaved-PARP (1:300 dilution) at 4 °C overnight. The tissue sections were then incubated with HRP-conjugated secondary antibodies for 1 h, followed by incubation of 3,3′-diaminobenzidine substrate (Thermo Fisher Scientific). For counterstaining, sections were stained with hematoxylin for 30 s and mounted with Aqua-Poly/Mount (Polyscience). Stained tissue slides were observed under an optical microscope (Axio imager A2; Carl Zeiss).

Finally, to confirm the apoptosis signaling pathway in HCC tumor tissues via western blotting, the separated tumor tissues were immersed in RIPA buffer (GenDEPOT) and ground using a tissue grinder (LMS Co., Ltd, Tokyo, Japan). Residual tissues were removed by centrifugation at 20,000*g* for 30 min at 4 °C. Equal amounts of protein lysates were electroporated on SDS-PAGE gels and transferred onto PVDF membranes (Millipore). The membranes were blocked with 5% skim milk (Sigma-Aldrich) and then incubated with specific primary antibodies and HRP-conjugated secondary antibodies (Santa Cruz Biotechnology). The blots were analyzed using the Amersham^TM^ Imager 6000 imaging system (GE Healthcare, Chicago, IL, USA). The ImageJ software (NIH) was used for the quantitative analysis of the western blots. The following antibodies were used: CD63 (1:1,000 dilution, Abcam); perforin, granzyme B (sc-8022), FasL (sc-33716), and TRAIL (1:1,000 dilution, Santa Cruz Biotechnology); caspase 3, caspase 7, caspase 8, caspase 9, cleaved-caspase 3, cleaved-caspase 7, cleaved-caspase 8, cleaved-caspase 9, PARP, cleaved-PARP, and β-actin (1:1000 dilution, Cell Signaling Technology).

### 2.14. Statistical analysis

All experiments were repeated at least three times, and the results are represented as the mean ± standard deviation (SD). Statistical analysis and graphical presentation were done using GraphPad Prism 9 (GraphPad Software, San Diego, CA, USA). More than two groups were compared using two-way analysis of variance (ANOVA) followed by Turkey’s post hoc test or Dunnett’s multiple comparison test. Significance levels are presented as *p < .05, **p < .01, ***p < .001, ^#^*p* < .05, ^##^*p* < .01, ^###^*p* < .001, and N.S. (no significance).

## 3. Results

### 3.1. Identification and characterization of NK-exo

**Figure 1.**
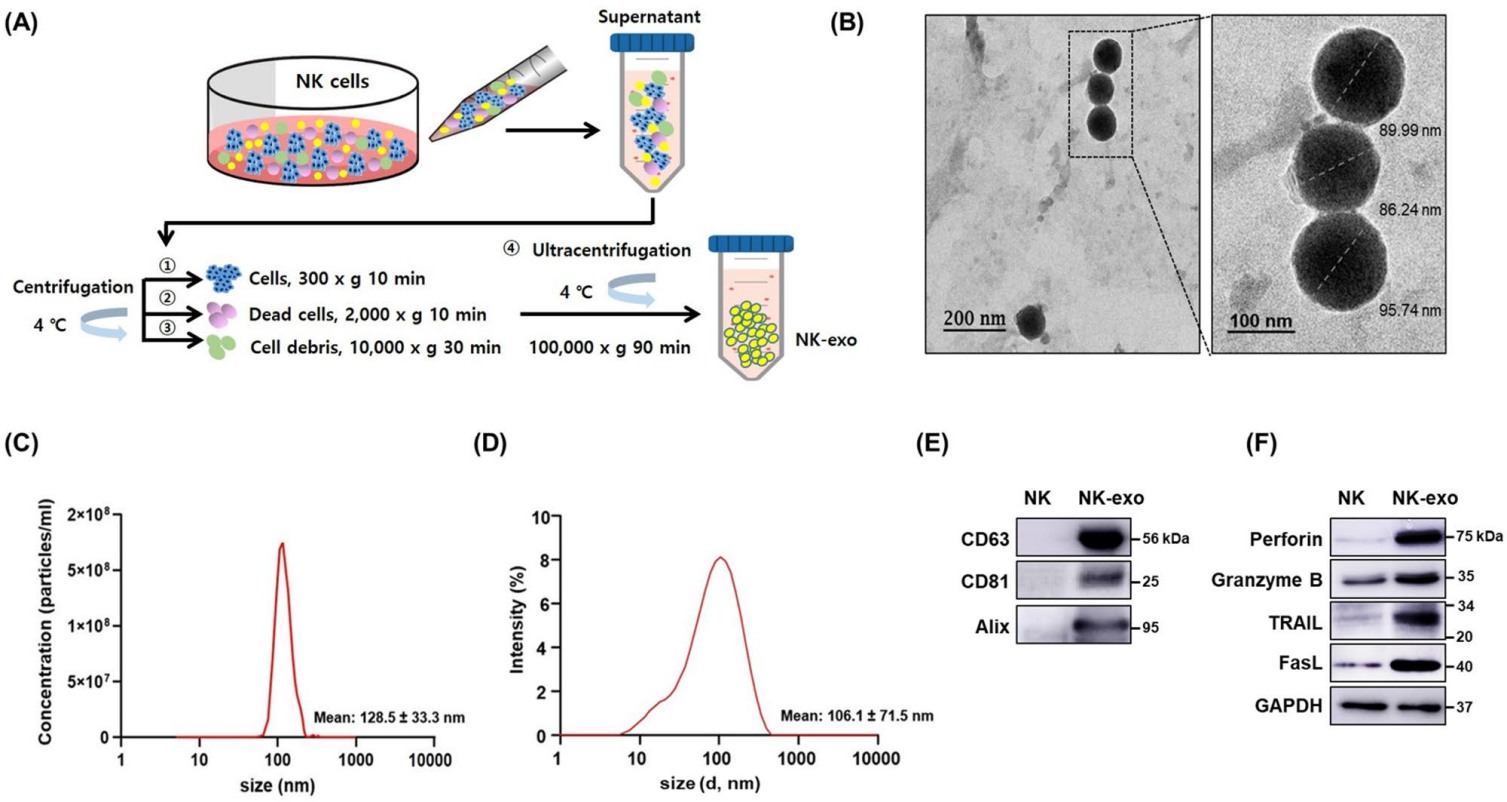
Isolation and characterization of NK-exo. A: Schematic depiction of the procedure for NK-exo isolation. NK-exo were isolated via ultracentrifugation. B: The morphology of isolated NK-exo was analyzed by TEM. Scale bars: 200 (left) and 100 nm (right). C and D: Size distributions of NK-exo were measured via NTA (C) and via DLS (D). E and F: The expression of exosome markers including CD63, CD81, and Alix (E) and cytotoxic proteins including perforin, granzyme B, TRAIL, and FasL (F) was evaluated using western blotting. GAPDH was used as a loading control. All data are representative of at least three independent experiments. NK, natural killer cells; NK-exo, NK cell-derived exosomes; TEM, transmission electron microscopy; NTA, nanoparticle tracking analysis; DLS, dynamic light scattering; FasL, Fas ligand; TRAIL, tumor necrosis factor-related apoptosis-inducing ligand.

The NK-exo were isolated by differential centrifugation (Figure 1(A)). They were then characterized via TEM, NTA, DLS, and western blotting. Exosomes are nanosized extracellular vesicles, 30–150 nm in diameter. The isolated NK-exo structures observed by TEM were ∼100 nm in size (Figure 1(B)) and obviously visualized the immunogold-labeled NK-exo (Supplementary Figure S1). The size distribution of the isolated NK-exo was determined by NTA (Figure 1(C)) and DLS (Figure 1(D)). Their sizes were assessed in terms of the peak diameters, which were found to be in the range of 100–130 nm (NTA: 128.5 ± 33.3 nm, DLS: 106.1 ± 71.5 nm). As shown in Figure 1(E), the isolated NK-exo were found to express specific exosome markers such as CD63, CD81, and Alix that are used for the characterization of exosomes. NK cells also release exosomes, which express typical cytotoxic protein markers (e.g. perforin, granzyme B, TRAIL, and FasL)-induced cell death (Fais, 2013). We confirmed the expression of cytotoxic protein markers (perforin, granzyme B, TRAIL, and FasL) in both NK-92 cells and NK-exo via western blotting. The expression of these proteins was found to be higher in NK-exo than in NK-92 cells (Figure 1(F)). Taken together, these results provide evidence for the successful isolation and characterization of NK-exo.

### 3.2. Uptake of NK-exo by HCC cells

**Figure 2.**
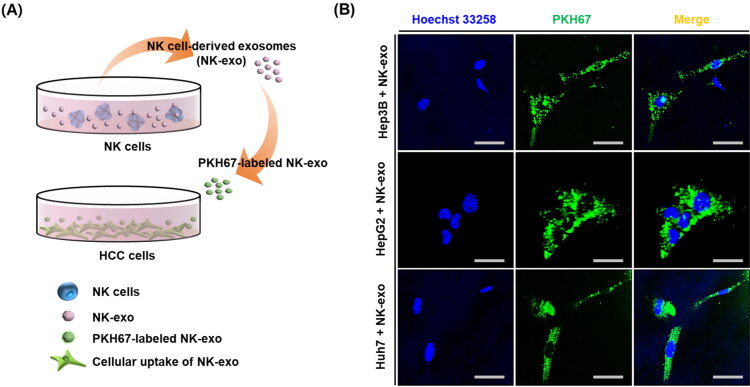
Uptake of NK-exo by HCC cells. A: Schematic representation of the experimental procedures for a green fluorescent dye, PKH67-labled NK-exo uptake by HCC cells. B: Hep3B, HepG2, and Huh7 cells were co-incubated with PKH67-labeled NK-exo (50 µg, green) for 10 h. The internalization of NK-exo was visualized via confocal laser scanning microscopy. Cell nuclei were stained with Hoechst 33258 (Blue). Scale bars: 50 μm.

Exosomes are taken up by target cells through several mechanisms, including endocytosis, receptor–ligand binding, and membrane fusion (Thery, 2011). Therefore, we evaluated the internalization of NK-exo into HCC cells, including Hep3B, HepG2, and Huh7. The green fluorescent dye, PKH67-labeled NK-exo for the evaluation of cellular uptake in HCC cells was schematically summarized in Figure 2(A). Green fluorescence was observed using a confocal laser scanning microscope to detect the cytoplasmic localization of PKH67-labeled NK-exo. NK-exo were found to be taken up by Hep3B, HepG2, and Huh7 cells (Figure 2(B)). These results suggest that NK-exo undergo internalization into HCC cells and are localized in the cytoplasm.

### 3.3. Nk-exo enhance the cytotoxicity of HCC cells

**Figure 3.**
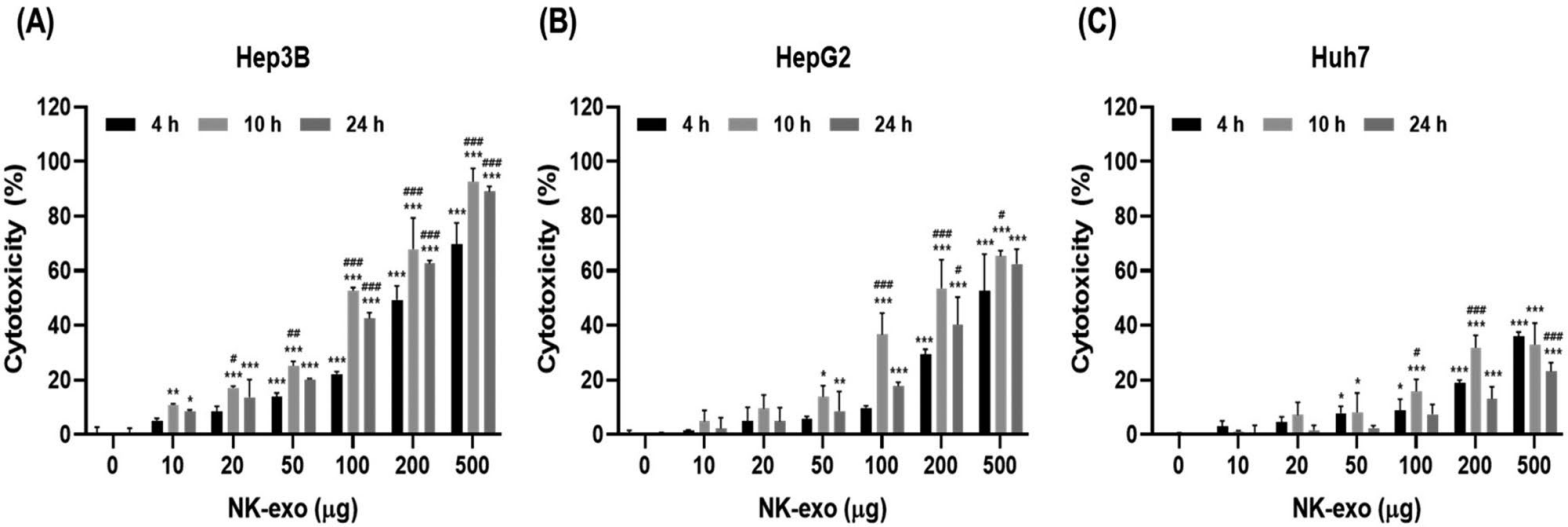
Cytotoxic effects of NK-exo in HCC cells. A–C: Hep3B (A), HepG2 (B), and Huh7 (C) cells were treated with different doses of NK-exo (0, 10, 20, 50, 100, 200, and 500 μg) for 4, 10, and 24 h. The cytotoxic activity of NK-exo in HCC cells was evaluated using LDH assay. All data are expressed as mean ± S.D. of at least three independent experiments. *p < .05, **p < .01, and ***p < .001 vs. the untreated control group; ^#^*p* < .05, ^##^*p* < .01, and ^###^*p* < .001 vs. the 4 h group. LDH, lactate dehydrogenase.

The functions of exosomes depend on the donor, parent cells (Thery et al., 2002; Raposo & Stoorvogel, 2013). NK cells regulate cell proliferation, survival, and apoptosis in primary liver cancers such as HCC (Liu et al., 2018; Juengpanich et al., 2019). We evaluated the cytotoxic activity of HCC cells (e.g. Hep3B, HepG2, and Huh7 cells) in different doses of NK-exo (0, 10, 20, 50, 100, 200, and 500 μg) at different times (4, 10, and 24 h) using LDH assay. Interestingly, NK-exo induced the highest cytotoxicity in Hep3B cells (Figure 3(A)), compared with HepG2 and Huh7 cells (Figure 3(B,C)) in a dose-dependent but not time-dependent manner, which was highest at 10 h. The highest cytotoxicity time of NK-exo in HCC cells has not been previously identified, the half maximal inhibitory concentrations (IC_50_) of NK-exo in Hep3B cells were calculated for the different times (Supplementary Figure S2). These results indicate that NK-exo exhibit significant cytotoxic activities against HCC cells.

### 3.4. Nk-exo reduce the cell viability of HCC cells

**Figure 4.**
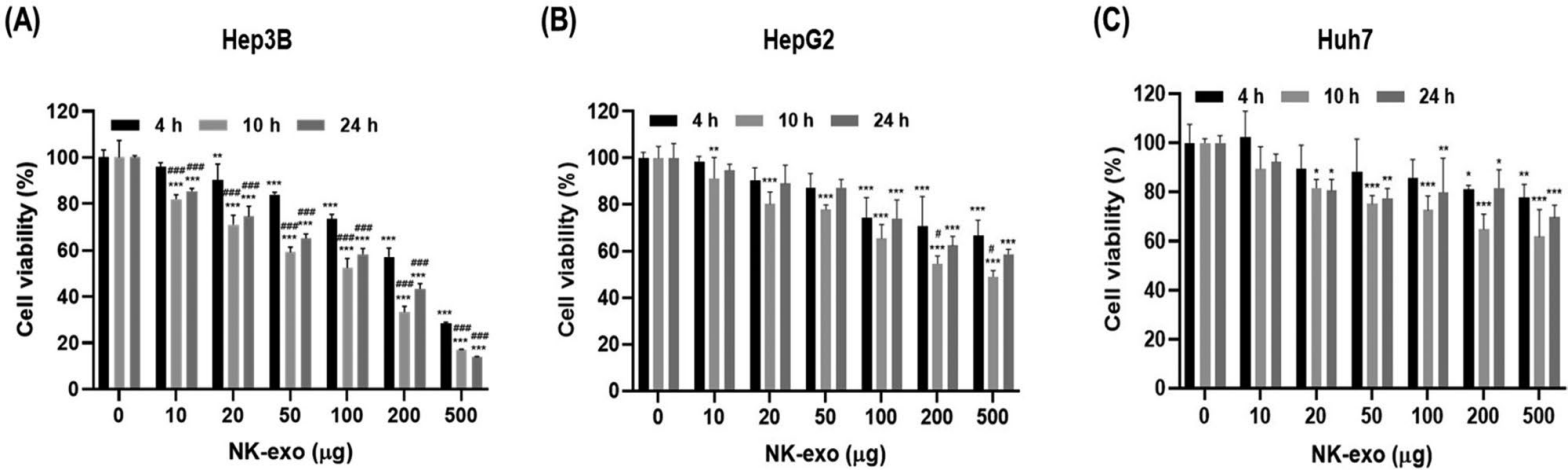
Cell viability effects of NK-exo in HCC cells. A–C: Hep3B (A), HepG2 (B), and Huh7 (C) cells were co-cultured with different doses (0, 10, 20, 50, 100, 200, and 500 μg) of NK-exo for 4, 10, and 24 h. The cell viability of NK-exos in HCC cells was measured using CCK-8 assay. All data are presented as the mean ± S.D. of at least three independent experiments. *p < .05, **p < .01, and ***p < .001 vs. the untreated control group; ^#^*p* < .05 and ^###^*p* < .001 vs. the 4 h group. CCK-8, cell counting kit-8.

Next, we evaluated the cell viability of NK-exo in HCC cells (e.g. Hep3B, HepG2, and Huh7 cells) in different doses of NK-exo (0, 10, 20, 50, 100, 200, and 500 μg) for different times (4, 10, and 24 h) using CCK-8 assay. As expected, NK-exo induced the highest cell viability reduction in Hep3B cells (Figure 4(A)), compared with HepG2 and Huh7 cells (Figure 4(B,C)) in a dose-dependent but not time-dependent manner, which was highest at 10 h. Optimal dose and treatment time are required for achieving high tumor killing by NK-exo. Taken together, these results suggest that NK-exo can significantly reduce cell viability in HCC cells, playing a critical role as anti-tumor effectors.

### 3.5. Nk-exo enhance the apoptosis of HCC cells

**Figure 5.**
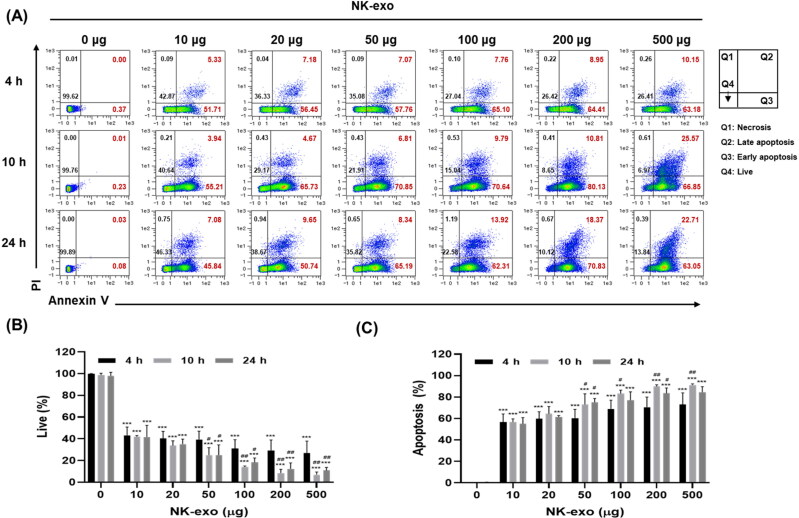
Apoptotic effects of NK-exo in HCC cells. A: Hep3B cells were co-cultured with different doses of NK-exo (0, 10, 20, 50, 100, 200, and 500 μg) for 4, 10, and 24 h, and then staining with Annexin V-FITC and PI. The apoptosis effects were analyzed using flow cytometry. B and C: Quantitation graphs of the viable (B) and apoptotic cells (C). All data are presented as mean ± S.D. of at least three independent experiments. ***p < .001 vs. the untreated control group; ^#^*p* < .05 and ^##^*p* < .01 vs. the 4 h group. PI, propidium iodide; FITC, fluorescein isothiocyanate.

In Figures 3 and 4, NK-exo induced the highest cytotoxicity and vability reduction in Hep3B cells at 10 h. Thus, we evaluated the apoptosis effects of NK-exos in Hep3B cells. Hep3B cells were co-cultured with different doses of NK-exo (0, 10, 20, 50, 100, 200, and 500 μg) for different times (4, 10, and 24 h) and then stained with Annexin V-FITC and PI. Apoptosis effects were evaluated using flow cytometric analysis (Figure 5(A)). We found that the number of viable Hep3B cells significantly decreased on exposure to NK-exo (Figure 5(B)). The proportion of early and late apoptotic Hep3B cells significantly increased on exposure to NK-exo in a dose-dependent but not time-dependent manner, which was highest at 10 h (Figure 5(C)). Notably, the apoptotic effects of NK-exo did not continue to increase for 24 h. These results demonstrate that NK-exo significantly enhance apoptosis in HCC cells.

### 3.6. Nk-exo do not affect the normal liver cells

**Figure 6.**
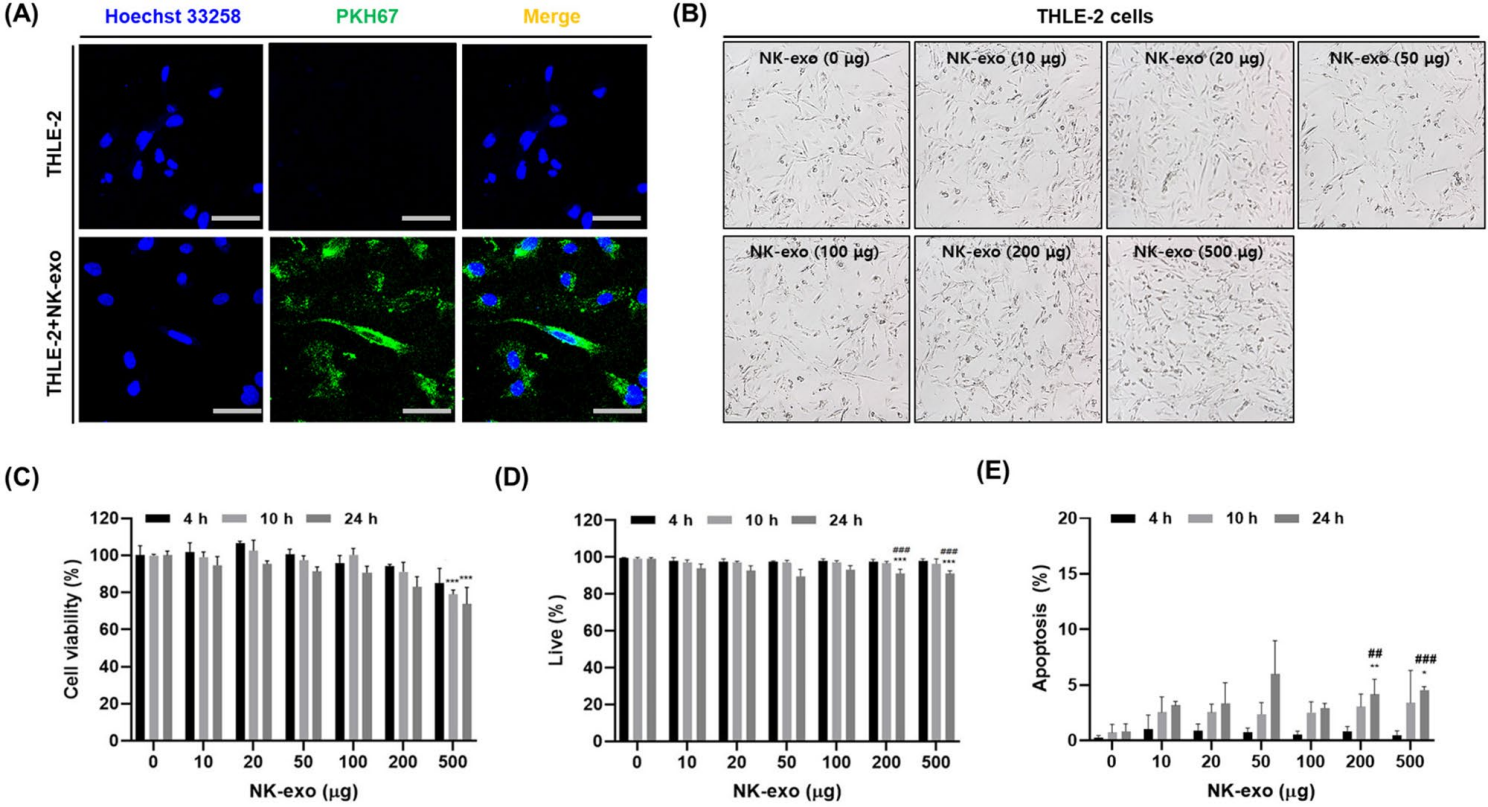
Cell viability and apoptotic effects of NK-exo in normal liver cells. A: THLE-2 cells were incubated with PKH67-labeled NK-exo (50 µg, green) for 10 h. NK-exo internalization was visualized by confocal laser scanning microscopy. Cell nuclei were stained with Hoechst 33258 (Blue). Scale bar: 50 μm. B: THLE-2 cells co-treated with different doses of NK-exo (0, 10, 20, 50, 100, 200, and 500 μg) for 10 h. Morphological changes were visualized via optical microscopy. C–E: THLE-2 cells-treated with different doses of NK-exo (0, 10, 20, 50, 100, 200, and 500 μg) for different durations (4, 10, and 24 h). C: Cell viability was measured using CCK-8 assay. D and E: The apoptosis was analyzed using flow cytometry with Annexin V-FITC and PI staining. Quantitation graphs of the viable (D) and apoptotic cells (E). All data are expressed as mean ± S.D. of at least three independent experiments. *p < .05, **p < .01, and ***p < .001 vs. the untreated control group; ^##^*p* < .01 and ^###^*p* < .001 vs. the 4 h groups. CCK-8, cell counting kit-8; PI, propidium iodide; FITC, fluorescein isothiocyanate.

As discussed in Figures 3–5, NK-exo were found to regulate cytotoxicity, cell viability, and apoptosis in HCC cells, including Hep3B, HepG2, and Huh7 cells. We also investigated the effects of NK-exo in normal liver cells. THLE-2 cells were co-incubated with PKH67-labeled NK-exo for 10 h. The uptake of NK-exo into THLE-2 cells was confirmed via confocal laser scanning microscopy (Figure 6(A)).

To evaluate the cytotoxic effects of NK-exo in normal liver cells, we assessed the morphology of THLE-2 cells via optical microscopy after exposure to different doses of NK-exo (0, 10, 20, 50, 100, 200, and 500 μg) for 10 h. NK-exo were not found to induce any significant morphological changes in THLE-2 cells (Figure 6(B)). Using CCK-8 assay, we also evaluated the effects of exposure to different doses of NK-exo (0, 10, 20, 50, 100, 200, and 500 μg) for different durations (4, 10, and 24 h) on the viability of THLE-2 cells. NK-exo did not significantly affect the viability of THLE-2 cells (Figure 6(C)).

To further verify the apoptotic effects of NK-exo in normal liver cells, THLE-2 cells were co-cultured with different doses of NK-exo (0, 10, 20, 50, 100, 200, and 500 μg) for different durations (4, 10, and 24 h). The percentages of viable and apoptotic THLE-2 cells were determined using flow cytometry analysis (Supplementary Figure S3). We found that the viable THLE-2 cells were not affected by NK-exo for different times (Figure 6(D)). The apoptotic THLE-2 cells were also not observed, suggesting that NK-exo did not induce apoptosis in THLE-2 cells (Figure 6(E)). These results indicate that NK-exo have no effect on the viability or apoptosis of normal liver cells.

### 3.7. Signaling mechanisms of NK-exo for HCC cell proliferation and apoptosis effects

Our results indicated that NK-exo regulate cytotoxicity, viability, and apoptosis in HCC cells (Figures 3–5). To determine the signaling mechanisms of HCC cell proliferation and apoptosis affected by NK-exo, we assessed the protein expressions that may be involved in these pathways using western blot analysis.

**Figure 7.**
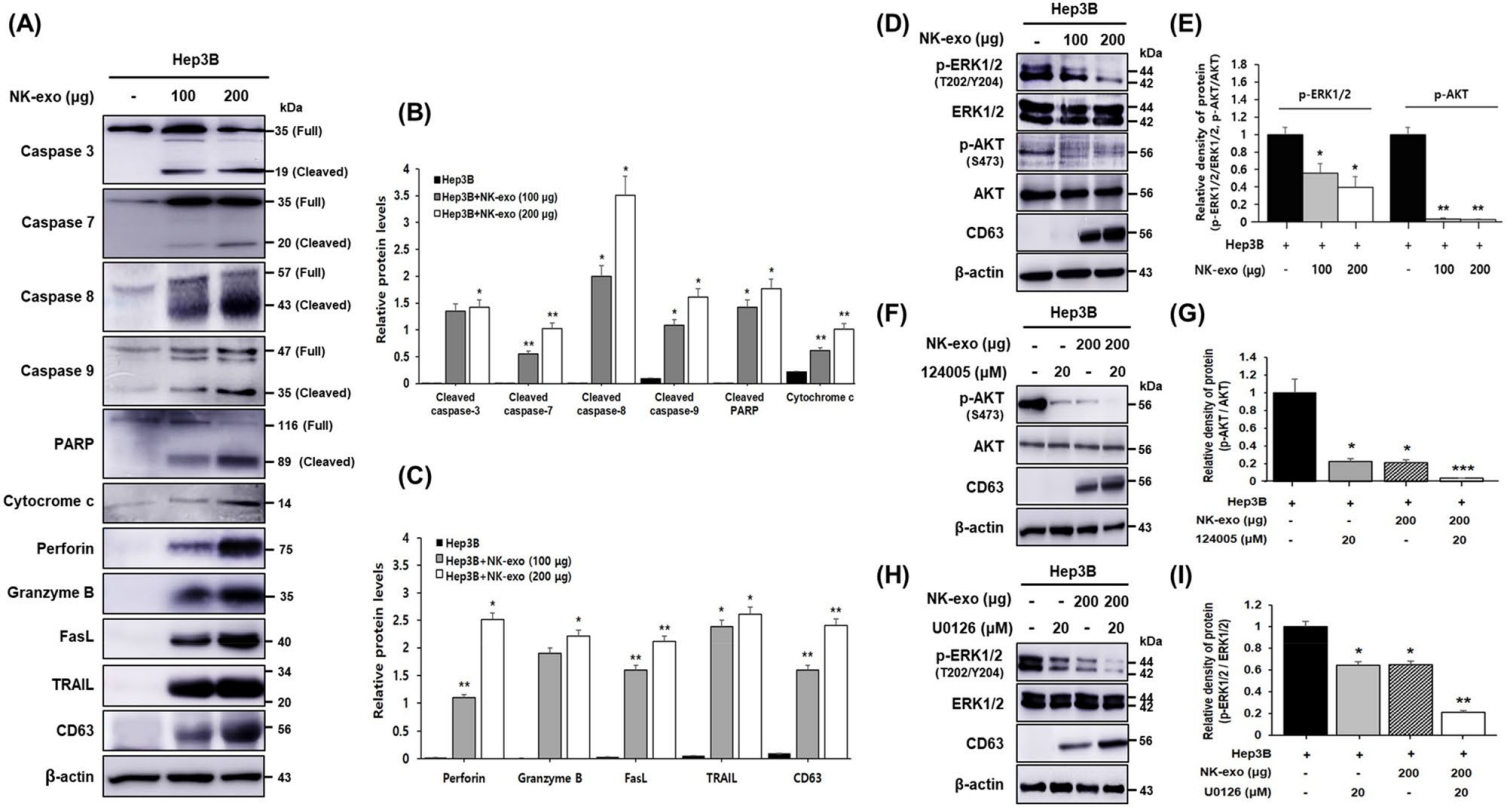
Signaling pathways for HCC cell proliferation and apoptosis affected by NK-exo. A: Hep3B cells were co-cultured with increased doses (100 and 200 μg) of NK-exo for 10 h. The expressions of specific apoptosis markers (caspase-3, -7, -8, -9, cytochrome c, and PARP) and cytotoxic proteins (perforin, granzyme B, FasL, and TRAIL) were analyzed using western blotting. Caspase activation occurs upon cleavage. B and C: The expression levels were quantified with ImageJ software, and normalized to that of β-actin. D: Hep3B cells were co-cultured with increased doses (100 and 200 μg) of NK-exo for 10 h. The phosphorylation of serine/threonine kinases (p-AKT and p-ERK1/2) was analyzed using western blotting. E: The histograms were quantified with ImageJ software, and the protein expressions were normalized to that of total proteins. F and H: Hep3B cells were co-cultured with NK-exo (200 μg), AKT inhibitor (124005, 20 μM), and ERK1/2 inhibitor (U0126, 20 μM) for 10 h. The inhibition of p-AKT at Ser473 (F) and p-ERK1/2 at Thr202/Tyr204 (H) were analyzed using western blotting. G and I: The histograms were quantified with ImageJ software, and the protein expressions were normalized to that of total proteins. All data are expressed as the mean ± S.D. of at least three experiments. *p < .05, **p < .01, and ***p < .001 vs. untreated Hep3B cells. CD63, as an exosomal marker and β-actin, as a loading control were used.

Apoptosis can be initiated through one of two pathways. In the intrinsic pathway, perforin (membrane disrupting protein) and granzyme B (serine protease) are secreted by exocytosis; they induce apoptosis via cytochrome c release (Metkar et al., 2002; Trapani & Smyth, 2002; Voskoboinik et al., 2006). In the extrinsic pathway, target cell death ligands (FasL and TRAIL) bind to their cognate receptors (Fas and TRAIL-R1/TRAIL-R2) (Zamai et al., 1998; Ferguson & Griffith, 2007). Both pathways involve the activation of apoptotic cysteine proteases (caspases) (Xu & Shi, 2007; Green & Llambi, 2015). In the intrinsic pathway, perforin and granzyme B induce the release of cytochrome c, which then activates caspase 9. In the extrinsic pathway, FasL and TRAIL activate caspase 8, followed by caspase 3 or caspase 7. This subsequently activates PARP in both pathways. As shown in Figure 5, NK-exo induced the apoptosis of Hep3B cells in a dose-dependent manner. Here, to evaluate the signaling pathway of apoptotic effects in Hep3B cells, we assessed the expressions of caspases and PARP using western blot analysis. Apoptosis effectors, caspases, and PARP activation are performed by cleavage. NK-exo increased the cleaved caspase (-3, -7, -8, and -9), cleaved PARP, and cytochrome c expression in a dose-dependent manner (Figure 7(A,B)). In addition, the cytotoxic proteins-induced apoptosis (e.g. perforin, granzyme B, FasL, and TRAIL) were increased in a dose-dependent manner (Figure 7(A,C)).

The AKT in the PI3K pathway and the ERK in the MAPK pathway control cell proliferation (Zhang & Liu, 2002; Chang et al., 2003; Cargnello & Roux, 2011; Manning & Toker, 2017). To determine the signaling mechanisms through which NK-exo inhibit cell proliferation in HCC cells, we assessed the expression levels of specific protein kinases (e.g. ERK1/2 and AKT) in Hep3B cells after exposure to NK-exo via western blot analysis. The phosphorylation of ERK1/2 at Thr202/Tyr204 (p-ERK1/2) and AKT at Ser473 (p-AKT) was found to be significantly decreased in Hep3B cells treated with NK-exo, compared with that in the untreated Hep3B cells. The decrease in phosphorylation showed dose-dependence (Figure 7(D,E)). The p-ERK1/2 and p-AKT in Hep3B cells were also found to be significantly suppressed by specific kinase inhibitors, AKT inhibitor (124005, 20 μM) (Figure 7(F,G)), and ERK1/2 inhibitor (U0126, 20 μM) (Figure 7(H,I)). Furthermore, the inhibition of p-AKT and p-ERK1/2 was synergistically enhanced by co-treatment with NK-exo and specific inhibitors. Taken together, we propose novel regulatory mechanisms of NK-exo in HCC cells, through modulation of serine/threonine kinase pathway-associated cell proliferation and caspase activation pathway-associated apoptosis.

### 3.8. Nk-exo promote in vivo tumor-targeting ability on HCC

**Figure 8.**
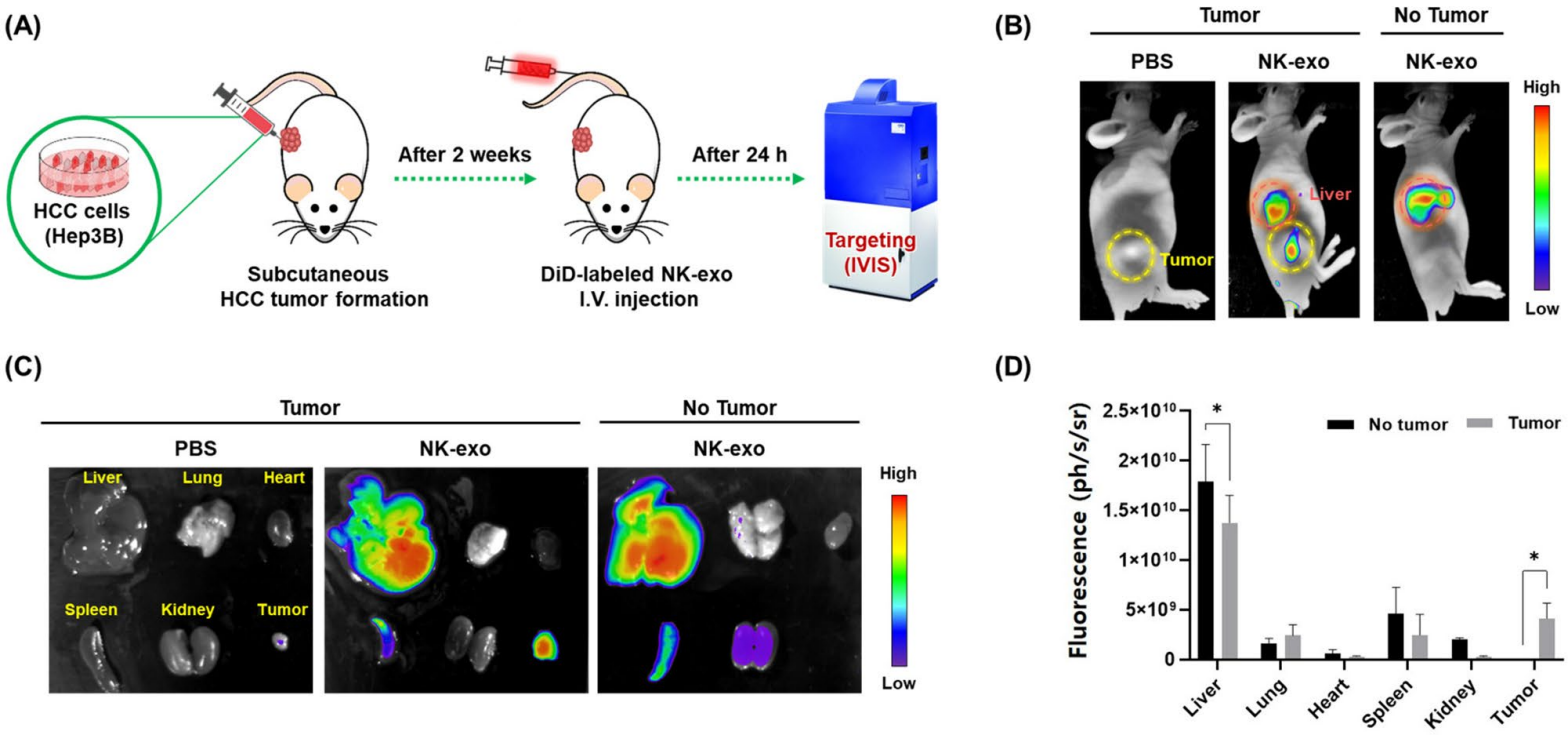
*In vivo* targeting ability of NK-exo in a subcutaneous HCC xenograft mouse model. A: Schematic depiction of the experimental protocols for the evaluation of the tumor-targeting ability of NK-exo in a subcutaneous xenografts mouse model of HCC. B and C: The mice were inoculated subcutaneously in the right back with 100 μL of Hep3B cells (1 × 10^7^ cells). After 2 weeks, no tumor and HCC-bearing mice were administered PBS (control) or DiD-labeled NK-exo (500 μg) via intravenous injection. After 24 h, the fluorescence imaging was monitored using the IVIS to NK-exo detection in the whole body (B), major organs such as the liver, lung, heart, spleen, kidneys, and *ex vivo* tumor (C). D: The fluorescent signals detected by IVIS in each organ and tumor were quantified. Data are expressed as mean ± S.D. from three independent experiments. *p < 0.05 vs. no tumor mice. IVIS, *in vivo* imaging system.

We evaluated the *in vitro* anti-tumor effects of NK-exo in Hep3B cells (Figures 3(A), 4(A), and 5). Previous studies have reported that exosomes secreted from cancers and immune cells travel to the specific tissues containing homing niches, and then have the strong targeting ability (Myint et al., 2020; Zhang et al., 2020). NK-EVs by IL-15 priming showed a tumor-homing ability in glioblastoma (Zhu et al., 2019). Thus, we investigated *in vivo* tumor-targeted homing ability of NK-exo using a subcutaneous HCC xenograft mouse model (Figure 8(A)). The fluorescent signals of injected DiD-labeled NK-exo (500 μg) were monitored in no tumor and HCC-bearing mice using the IVIS imaging system (Figure 8(B)). Mice imaging showed that NK-exo targeted the tumor (yellow circle) and liver (red circle) (Figure 8(B), middle panel). Furthermore, to evaluate the targeting efficiency, the major organs (the liver, lung, heart, spleen, and kidneys) and tumor were harvested for *ex vivo* imaging, and then also visualized the fluorescent signals using the IVIS imaging system (Figure 8(C)). NK-exo exhibited significantly high uptake and accumulation in the liver and target tumor in HCC-bearing mice (Figure 8(C), middle panel). Quantitative analysis showed that HCC-bearing mice exhibited complete tumor uptake of NK-exo and relatively reduced liver uptake of NK-exo, compared with no tumor mice (Figure 8(D)). Our results suggest that NK-exo exhibit the strong tumor-targeted homing efficacy, which is important for HCC therapy.

### 3.9. Nk-exo improve in vivo anti-tumor effects on HCC

**Figure 9.**
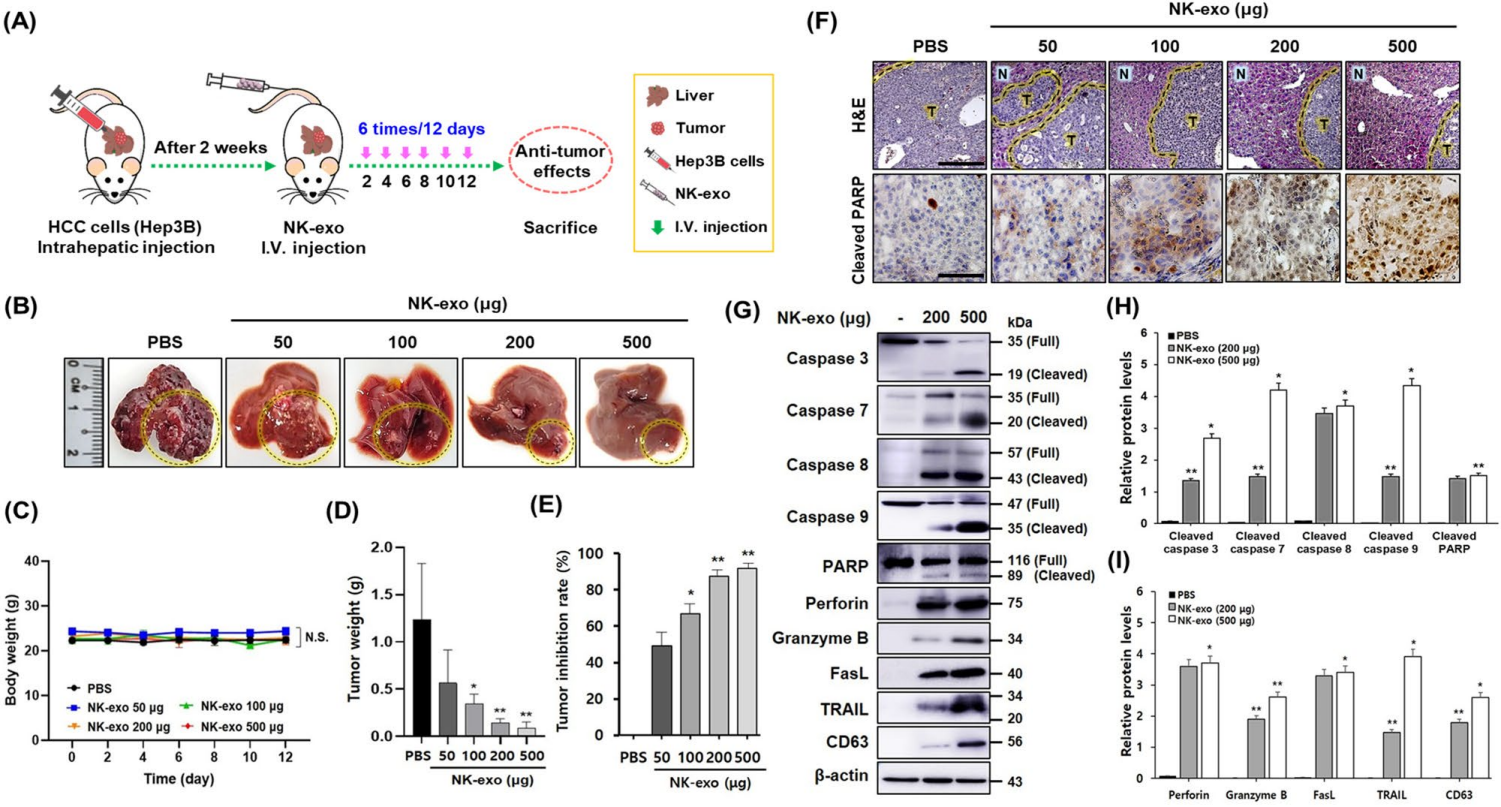
*In vivo* anti-tumor effects of NK-exo in an orthotopic HCC xenograft mouse model. A: Schematic depiction of the experimental protocol for the evaluation of NK-exo targeting ability in orthotopic xenograft mouse models of HCC. 50 μL of Hep3B cells (2 × 10^6^ cells) were inoculated directly into the livers of mice. After 2 weeks, the HCC-bearing mice were administered PBS (control) or different doses of NK-exo via intravenous injection, once every 2 days for 12 days. The HCC-bearing mice were sacrificed and then the anti-tumor effects in each group were assessed. B: Representative images of a dissected liver from HCC-bearing mice treated with PBS (control) or NK-exo (50, 100, 200, and 500 μg) are shown. The yellow-dotted circles indicate tumors. C–E: Changes in body weight (C), tumor weight (D), and tumor inhibition rate (E) of HCC-bearing mice treated with PBS (control) or NK-exo (50, 100, 200, and 500 μg) are shown. Body weight was measured until 12 days. Tumor weight was determined after being harvested from the end of experiment. The tumor inhibition rate was calculated as: [1 – (TW_NK-exo treated_/TW_PBS control_)] × 100 (%). All data are presented as mean ± S.D. of at least three independent experiments. *p < .05, **p < .01, and N.S. (no significance) vs. PBS control group. F: Representative histologic images of H&E staining (top panel) and IHC staining (bottom panel) against apoptosis marker cleaved PARP in tumor tissues of HCC-bearing mice treated with PBS (control) or NK-exo (50, 100, 200, and 500 μg). Yellow-dotted lines indicate the boundary of normal tissues (N) and tumor tissues (T). Scale bars: 200 μm (top panel) and 50 μm (bottom panel). G: The apoptosis signaling pathway in tumor tissues of HCC-bearing mice treated with NK-exo (200 and 500 μg) was evaluated. The expressions of specific apoptotic makers (caspase-3, -7, -8, -9, and PARP) and cytotoxic proteins (perforin, granzyme B, FasL, and TRAIL) were analyzed using western blotting. Caspase activation occurs upon cleavage. CD63, as an exosome marker and β-actin, as a loading control were used. H and I: The histograms were quantified with ImageJ software, and normalized to that of β-actin. All data are presented as mean ± S.D. of at least three independent measurements. *p < .05 and **p < .01 vs. untreated NK-exo group. TW_PBS control_, tumor weight of PBS control group; TW_NK-exo treated_, tumor weight of NK-exo treated group; H&E, hematoxylin and eosin; IHC, immunohistochemistry.

To evaluate the *in vivo* therapeutic effects of NK-exo in HCC, we established an orthotopic xenograft mouse model (Figure 9(A)). We investigated that NK-exo can act as a cytotoxic regulator to exhibit anti-tumor activity. We found that NK-exo exhibited significant improvements in therapeutic effects of HCC (yellow circles) compared with PBS control (Figure 9(B)). The therapeutic effects of NK-exo (500 μg) were similar to normal liver. No significant differences on body weights were observed (Figure 9(C)). The tumor growth was described by tumor weight in each group. Tumor treated with NK-exo was more inhibited in growth than PBS control by reducing tumor weight (Figure 9(D)). Tumor inhibition rate was also increased in a dose-dependent manner (Figure 9(E)). Taken together, these results demonstrated that NK-exo induced the significant suppression of HCC tumor growth, providing support for the therapeutic effect.

In addition, H&E staining and IHC staining against cleaved PARP (apoptosis marker) were conducted to evaluate the further toxicity of NK-exo in hepatic tumor tissues of HCC-bearing mice (Figure 9(F)). H&E staining showed a greater degree of tumor apoptosis in the tumor group-treated NK-exo than in the PBS control group (Figure 9(F), top panel). IHC staining showed that the expression levels of cleaved PARP were considerably increased in the hepatic tumor tissue treated with NK-exo (500 μg), compared with that in the PBS control group (Figure 9(F), bottom panel).

Finally, we then evaluated the apoptosis signaling pathway responsible for inducing cell death in tumor tissues (Figure 9(G–I)). Two major apoptosis pathways are necessary for regulating cell death in cancers: the intrinsic pathway, involving perforin and granzyme B (Voskoboinik et al., 2006), and the extrinsic pathway, involving FasL and TRAIL (Leite-de-Moraes et al., 2000; Ferguson & Griffith, 2007). Both pathways initiate and depend on the activation of apoptotic caspases (Xu & Shi, 2007; Green & Llambi, 2015). We evaluated the expression of major apoptotic markers (caspase-3, -7, -8, -9, and PARP) using western blot analysis. NK-exo significantly enhanced *in vivo* apoptosis in HCC tumor tissues by increasing the activation of caspase (-3, -7, -8, -9) and PARP in a dose-dependent manner (Figure 9(G,H)). In addition, the expression levels of the cytotoxic proteins (perforin, granzyme B, FasL, and TRAIL) were also increased in a dose-dependent manner (Figure 9(G,I)). Quantitative analysis revealed that, increased NK-exo (200 μg and 500 μg) enhanced cleaved caspase (-3, -7, -8, -9) and cleaved PARP (Figure 9(H,I)). Our results demonstrate that NK-exo exhibit strong *in vivo* anti-tumor effects through the intrinsic and extrinsic apoptotic pathways-induced tumor suppression.

## 4. Discussion

Exosomes can mediate intercellular communication to regulate the biological processes in health and disease, including tumorigenesis and tumor metastasis (Iero et al., 2008). Immune cell-derived exosomes such as NK-exo can regulate cancer progression and metastasis. They have applications in cancer diagnosis and immunotherapy. Previous studies reported that NK-exo acts as an anti-tumor effector via regulating cytotoxicity and apoptosis kills human cancers, including pancreatic cancer (Sun et al., 2019), leukemia (Lugini et al., 2012; Di Pace et al., 2020), neuroblastoma (Shoae-Hassani et al., 2017; Neviani et al., 2019; Wang et al., 2019), melanoma (Zhu et al., 2017), breast cancer (Kaban et al., 2021), and lung cancer (Kang et al., 2021). However, the biological functions of NK-exo in solid tumors, such as liver cancer, especially HCC remain unclear.

NK cells regulate cellular cytotoxic activity and cytokine production through cytotoxic proteins (e.g. perforin, granzyme B, FasL, and TRAIL), which are important participants in the controlling tumor progression. Nonetheless, NK cells have a lot of limitations in an anti-tumor response of cancer therapy. First, the poor ability of NK cells to reach tumor tissues limits their application to the treatment of solid tumors (Melero et al., 2014). Second, the tumor microenvironments, including hypoxia, lack of nutrition, and nonappropriate metabolic conditions remain a major barrier to the effectiveness of transferring from NK cells to cancer cells (Bagheri et al., 2021). Acidic tumor microenvironments prevent Fas/FasL interactions and decrease perforin/granzyme B release from NK cells (Fischer et al., 2000). These problems stimulate tumor progression and reduce therapeutic responses. Interestingly, exosomes are not significantly affected by acidic tumor microenvironments (Parolini et al., 2009; Boussadia et al., 2018). NK-exo can more easily extravasate into the tumor microenvironment, and the low tumoral pH may better facilitate NK-exo fusion with cancer cells. NK-exo provide an environment in which tumor-killing effector molecules can be delivered directly to the tumor site and thus overcome the homing deficiency of NK cells. Hence, NK-exo have gained the spotlight for their potential clinical applications in cancer therapy.

In the present study, we first demonstrated that NK-exo play a critical role for enhancing the tumor-targeting and anti-tumor activity of HCC, both *in vitro* and *in vivo*. NK-exo do not require many reagents for the activity of NK-92 cells and have the advantage to isolate very easily and quickly, compared to Peripheral blood mononuclear cell (PBMC) used in many previous studies (Supplementary Table S1). We comfirmed that the characterizations, including morphology and size of NK-exo were revealed by TEM (Figure 1(B) and Supplementary Figure S1), NTA (Figure 1(C)), and DLS (Figure 1(D)) in the size range of 80–130 nm. Notably, NK-exo contain specific cytotoxic proteins (involving perforin, granzyme B, FasL, and TRAIL), originating from NK cells and expressed higher in NK-exo than those in NK cells (Figure 1(F)). The cytotoxic proteins are responsible for regulating anti-tumor activities of target cells, such as cell survival, proliferation, and apoptosis in tumorigenesis (Lugini et al., 2012; Fais, 2013; Jong et al., 2017; Zhu et al., 2017). Here, we demonstrated that NK-exo enhanced *in vitro* anti-tumor effects, including cytotoxicity (Figure 3), cell viability reduction (Figure 4), and apoptosis (Figure 5) on HCC cells. Interestingly, NK-exo induced the maximal cell viability, cytotoxicity, and apoptosis in Hep3B cells at 10 h, compared with HepG2 and Huh7 cells. The highest cytotoxicity time of NK-exo in HCC cells has not been previously identified, but we first reported in this study. Importantly, NK-exo did not show significant cytotoxicity and apoptosis in normal liver THLE-2 cells (Figure 6). Taken together, NK-exo can be a suggestion as an immunotherapeutic tool for HCC.

**Figure 10.**
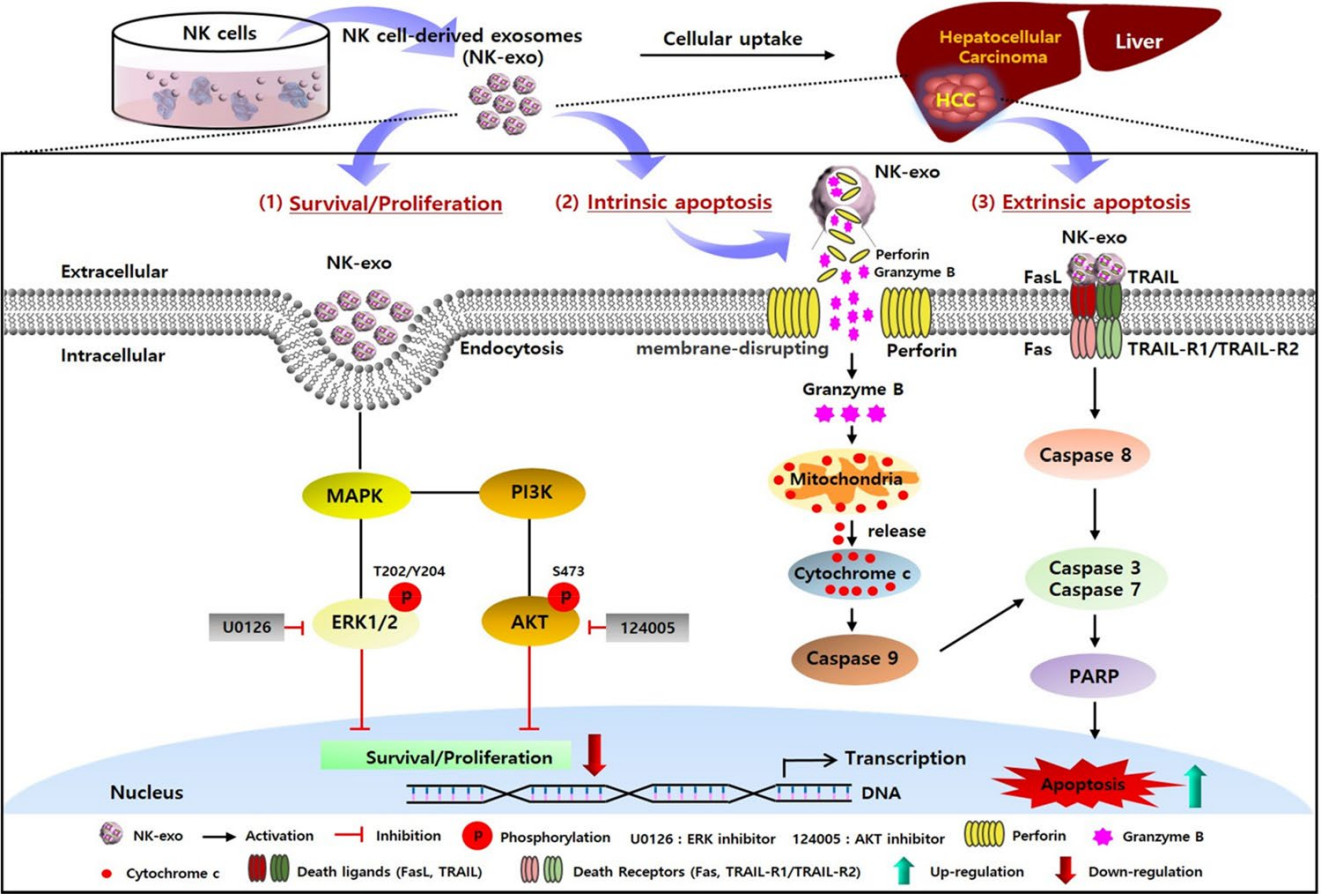
Schematic depiction of the mechanisms through which NK-exo affect the survival, proliferation, and apoptosis of HCC to exhibit anti-tumor effects. (1) Survival/Proliferation pathway: NK-exo downregulate the activation of ERK1/2 in the MAPK pathway and that of AKT in the PI3K pathway, via inhibiting phosphorylation of AKT at Ser473 and ERK1/2 at Thr202/Tyr204. (2) Intrinsic apoptosis pathway: NK-exo initiate the pathway by perforin/granzyme B release and mediate cytochrome c release for the downstream activation of caspase 9. (3) Extrinsic apoptosis pathway: NK-exo initiate the pathway by death ligands (FasL and TRAIL) that bind to death receptors (Fas and TRAIL-R1/TRAIL-R2). Downstream signaling activates the initiator caspase 8. The two apoptosis pathways converge with caspase 3 or caspase 7 activation, and then finally induce apoptosis via PARP activation.

However, the underlying mechanisms of HCC proliferation and apoptosis mediated by NK-exo are poorly understood, compared to many previous studies (Supplementary Table S1). Here, the specific anti-tumor mechanisms of NK-exo in HCC were first demonstrated and summarized in Figure 10. There are two main apoptotic pathways: the intrinsic pathway involving perforin and granzyme B, which trigger the release of mitochondria-mediated cytochrome c, leads to the activation of caspase 9; the extrinsic pathway involving FasL and TRAIL, which bind to their cognate receptors and trigger the activation of initiator caspase 8 (Warren & Smyth, 1999; Voskoboinik et al., 2006). The two apoptosis pathways converge for the activation of caspase 3 or caspase 7, ultimately inducing apoptosis via activation of PARP. Thus, we assessed the signaling mechanisms of HCC apoptosis through the protein expressions involved in these pathways using western blot analysis. We first demonstrated that NK-exo induced apoptosis in Hep3B cells by increasing the activation levels of cleaved caspase (-3, -7, -8, and -9), cleaved PARP, and cytotoxic proteins (perforin, granzyme B, FasL, and TRAIL) in a dose-dependent manner (Figure 7(A–C)). NK-exo can act as an apoptosis regulator on HCC via enhancing the activation of caspases-dependent apoptosis in both intrinsic and extrinsic pathways. In addition, NK cells have been reported to regulate tumor cell survival and proliferation through the ERK1/2 in the MAPK pathway and the AKT in the PI3K pathway (Chow et al., 2005; Nishimura et al., 2009; Yang et al., 2017). Previous studies have reported that NK-exo exhibit anti-tumor effects by inhibiting tumor proliferation, which are decreased by p-ERK1/2 and p-p38 signaling on melanoma (Zhu et al., 2017). However, the potential cell survival and proliferation pathway of NK-exo in HCC are still unclear. Thus, to determine the underlying mechanisms of NK-exo activity-induced HCC cell proliferation, we evaluated the roles of AKT in the PI3K pathway and those of ERK1/2 in the MAPK pathway using western blot analysis. We found that NK-exo downregulate cell survival and proliferation by inhibiting the phosphorylation of AKT at Ser473 and ERK1/2 at Thr202/Tyr204 in Hep3B cells (Figure 7(D,E)). Moreover, the specific AKT inhibitor (124005; Figure 7(F,G)) and ERK1/2 inhibitor (U0126; Figure 7(H,I)) significantly inhibited p-ERK1/2 and p-AKT levels. Notably, the inhibitory effects of these kinase inhibitors were more synergistically increased, co-treated with NK-exo (Figure 7(F–I)). We further suggested that NK-exo can downregulate HCC cell proliferation through inhibitory phosphorylation of two serine/threonine kinase pathways. Taken together, our findings provide novel insight into the underlying mechanisms of NK-exo in HCC killing effects.

*In vivo* biodistributions are essential and important for demonstrating the anti-tumor functions and therapeutic applications of NK-exo. Exosomes secreted from immune cells have the strong targeting and homing ability (Myint et al., 2020; Zhang et al., 2020). This study provides the first evidence in tumor-targeting ability and anti-tumor effects of NK-exo using the orthotopic and subcutaneous HCC mouse models. NK-exo were found to exhibit strong tumor-targeted homing ability in a subcutaneous HCC-bearing mice (Figure 8(B)). Notably, NK-exo showed higher uptake and accumulation in the liver and tumor than in the other organs (Figure 8(C,D)). Our findings can suggest a solution to the low targeting efficiency of NK cells. NK-exo does not affect the apoptosis of normal liver, THLE-2 cells (Figure 6). The possibility of NK-exo causing apoptosis in no tumor such as major organs (e.g. liver, lung, heart, spleen, and kidneys) may be impossible *in vivo*. In addition, *in vivo* anti-tumor effects of NK-exo in HCC were assessed using an orthotopic mouse model (Figure 9(A)). No studies have been conducted in HCC cancer therapies of NK-exo using an orthotopic xenograft model, as shown in Supplementary Table S1. We confirmed that NK-exo exhibit significant therapeutic activity in a dose-dependent manner (Figure 9(B)), and markedly inhibited the tumor growth (Figure 9(D,E)). In HCC tumor tissues, NK-exo significantly enhanced apoptosis through the activation of caspase (-3, -7, -8, and -9), PARP, and cytotoxic proteins (perforin, granszyme B, FasL, and TRAIL) in a dose-dependent manner (Figure 9(G–I)). Taken together, NK-exo exhibited strong tumor-homing ability and anti-tumor effects in HCC, at least in part, by increasing the activation of caspases-dependent apoptosis pathway.

Here, we confirmed that NK-exo may be a candidate for immunotherapy in solid tumors such as HCC, which can overcome the disadvantages of NK cells. Nevertheless, many studies on NK-exo are still remained. First, exosomes-based delivery system has particular benefits such as specificity, stability, and safety. Their homing characteristic, exosomes can deliver their cargo to specific targets over a long distance. NK-exo may be used as a carrier in drug delivery systems (DDS) to transport other anti-cancer drugs (e.g. paclitaxel, doxorubicin, and sorafenib) or chemicals to the target cells, organs, and tissues through the high loading efficiency for treating various diseases. Second, exosomes can contain many types of biomolecules, including proteins, lipids, and nucleic acids. Bioengineered molecules may help improve the therapeutic effects of NK-exo. Third, the cytokines play a critical role in immune regulation and have been approved for therapeutic applications in cancer therapy. Previous study reported that NK-EV by IL-15 priming enhanced anti-tumor potency in glioblastoma (Zhu et al., 2019). However, the various cytokine effects on the biogenesis of NK-exo are not well known. Finally, the mechanisms of how NK-exo recognize tumor cells or activated immune cells, and exhibit specific effects are still unclear and need further research.

## 5. Conclusions

In summary, the present study provided the first evidence that NK-exo are an innovative nano-therapeutic candidate, which can overcome several disadvantages of NK cells via enhancing the high tumor-targeting ability and potent anti-tumor effects. Therefore, NK-exo can improve the clinical applicability and therapeutic benefits in the treatment of solid tumors, especially HCC.

## Data Availability

The data that support the findings of this study are available from the corresponding author upon reasonable request
